# Administration of *Ligilactobacillus salivarius* MP101 in an Elderly Nursing Home during the COVID-19 Pandemic: Immunological and Nutritional Impact

**DOI:** 10.3390/foods10092149

**Published:** 2021-09-11

**Authors:** Marta Mozota, Irma Castro, Natalia Gómez-Torres, Rebeca Arroyo, Yolanda Lailla, Mario Somada, Claudio Alba, Juan Miguel Rodríguez

**Affiliations:** 1 Department of Nutrition and Food Science, Complutense University of Madrid, 28040 Madrid, Spain; martamoz@ucm.es (M.M.); irmacastro@ucm.es (I.C.); natgom07@ucm.es (N.G.-T.); rebecaa@vet.ucm.es (R.A.); claudioalbarubio@gmail.com (C.A.); 2 Villa Villera, 22142 Sieso de Huesca, Spain; yolanda@villavillera.es (Y.L.); mario@villavillera.es (M.S.)

**Keywords:** probiotics, *Ligilactobacillus salivarius*, elderly, nursing home, COVID-19

## Abstract

The elderly population living in nursing homes is particularly vulnerable to COVID-19 although individual susceptibility to SARS-CoV-2 infection may be related to the host microbiota. The objective of this work was to investigate the effect of *Ligilactobacillus salivarius* MP101 on the functional (Barthel index), cognitive (GDS/FAST), and nutritional (MNA) status as well as on the nasal and fecal inflammatory profiles of elderly residents living in a nursing home that is highly affected by COVID-19. A total of 25 residents participated in the trial, which involved the daily ingestion of a dairy product (*L. salivarius* MP101: 9.3 log_10_ CFU per unit) for 4 months. Nasal and fecal samples were analyzed for 37 immune factors at recruitment and at the end of the study. After the trial, no change in the GDS/FAST scores were found but, in contrast, the values for the Barthel index and the MNA score improved significantly. The concentrations of some immune factors changed significantly after the trial, including a decrease in the concentrations of BAFF/TNFSF13B, APRIL/TNFSF13, IL8, IL31, osteopontin, sTNF-R1, and sTNF-R2, and an increase in chitinase 3-like 1, IL19, IL35, and pentraxin 3 was also observed. In conclusion, *L. salivarius* MP101 seems to be a promising strain for improving or maintaining health in this highly vulnerable population.

## 1. Introduction

Severe Acute Respiratory Syndrome Coronavirus 2, universally known as SARS-CoV-2, was first identified in 2019 as the causative agent of a new acute respiratory disease (coronavirus disease 2019; COVID-19), which was recognized as a pandemic by the World Health Organization on 11 March 2020. It has affected virtually all countries around the world and has caused several millions of deaths. The severity of the disease is higher among people aged ≥60 years; people with underlying health conditions, such as hypertension, diabetes, obesity, cardiovascular disease, chronic respiratory disease and weakened immune systems; and/or those living in long-term care facilities. However, most of those infected remain asymptomatic, a fact that complicates the control of the disease.

The fact that SARS-CoV-2 initially interacts with the mucosal epithelia associated with a complex microbiota (naso-oro-pharyngeal cavities) and that its mechanism of infection is related to the angiotensin-converting enzyme receptor 2 (ACE2), whose activity is influenced and, in turn, influences the microbiota of the upper respiratory and gastrointestinal tracts [1,2], suggests an implication of the microbiota in individual susceptibility to COVID-19 and in the severity of the infection [3,4]. The modulatory role of the respiratory microbiota has already been observed with other respiratory viruses, including other coronaviruses and the respiratory syncytial virus [5,6,7]. In this sense, it has been postulated that people with “eubiotic” respiratory and gastrointestinal microbiotas would be more likely to have an asymptomatic or mild SARS-CoV-2 infection given that their microbiotas are associated with appropriate mucosal immune responses. On the contrary, people with respiratory or intestinal dysbiosis would not be able to develop the correct responses and would be more prone to a more severe infection and to complications (including cytokine storm, bacterial and fungal secondary infections, and sepsis) [8,9].

Given that the composition of the microbiota is relevant to our health and that the COVID-19 frame does not seem to be an exception, various articles have postulated the application of strategies stimulating the presence of those members of the respiratory and/or digestive microbiota with beneficial functions for our health and enhancing the mucosal barriers and immune function, such as probiotics [10]. However, most of them simply address/deal with the benefits of probiotics for general health or speculate about the possible effects that probiotics or some of their metabolites could have on patients who suffer from this infection [11,12,13]. In contrast, data from clinical trials specifically designed for the prevention or as an aid in the treatment of COVID-19 through the use of probiotics are very scarce and are limited to hospitalized COVID-19 patients [14,15].

In any case, COVID-19 contains numerous aspects that constitute clear targets for the application of probiotics [16] including (a) the management of diarrhea and other digestive symptoms that are widespread among patients, (b) the modulation of the immune and inflammatory response to the virus, (c) the prevention of bacterial or fungal co-infections, (d) the prevention or treatment of microbiota alterations associated with the use of respirators, corticosteroids, antibiotics, and antifungals, (e) the pre-existing situation of dysbiosis that usually characterizes the population most vulnerable to COVID-19 (e.g., elderly, diabetic, immunosuppressed), which can also be a consequence of the treatments received, (f) the rise in cases of anxiety, depression, or stress associated with the pandemic, and (g) the enhancement of vaccine responses due to adjuvant effects.

In this context, the objective of this work was to investigate the effect of a probiotic strain on the functional, cognitive, and nutritional status as well as on the nasal and fecal inflammatory profiles of patients living in an elderly nursing home that is highly affected by COVID-19.

## 2. Materials and Methods

### 2.1. Selection of the Probiotic Strain

The probiotic strain used in this trial (*Ligilactobacillus salivarius* MP101) was initially isolated from the feces of a healthy breastfed infant. It was selected as the best candidate for a trial aimed to the prevention or treatment of bronchiolitis caused by the respiratory syncytial virus (RSV) in the framework of a previous study on the impact of the nasal and fecal microbiotas on the severity and outcome of RSV-associated bronchiolitis [17]. However, the rapid spread and dramatic impact of the COVID-19 pandemic led us to change our initial target temporarily, from RSV-associated bronchiolitis to COVID-19, to try to minimize the impact of the latter infection in a nursing home for the elderly. Both SARS-CoV-2 and RSV share some features: they are syncytial viruses that enter the host through the mucosal surface of the upper respiratory tract and cause acute respiratory infections, the severity of which may be modulated, among other factors, by the host microbiota. 

### 2.2. Production and Quality Control of the Study Product

In an initial meeting, the director of the elderly nursing home informed us that, if possible, the probiotic strain should be administered as a dairy (yogurt-like) product since all residents received a yogurt during dinner. Any other format (capsules or sachets) would require an individual assessment of product intake, which would imply management problems derived from the reduced available staff when the trial was planned (a high proportion of the staff was in quarantine due to being SARS-CoV-2-positive).

As a consequence, a dairy product containing ≥9 log_10_ colony-forming units (CFU) of *L. salivarius* MP101 per product unit (125 g) was developed. For this purpose, 50 g of the freeze-dried strain (~9.3 log_10_ CFU/g) were used to inoculate 100 L (a batch) of milk that had previously been pasteurized (85 °C, 30 min) and cooled to 36 °C. Yogurt starter cultures were not added. The inoculated milk was homogenized by stirring for 1 h and it was then aseptically distributed in 125 g containers, which were fermented at 36 °C for 15 h. The pH values of at least two containers were monitored during each batch fermentation. After fermentation, the containers were stored and distributed at refrigeration temperature (2–8 °C). The manufacture of the probiotic dairy product was conducted at the facilities of Villa Villera (Sieso de Huesca, Huesca, Spain), a dairy company that altruistically provided the milk and all of the the necessary material (containers) and processing (and packaging) equipment.

Four containers from each batch were randomly selected to assess the concentration and purity of the strain at day 1 after production. For this purpose, the content of each container was homogenized, and the homogenized product (5 g) was submitted to serial decimal dilutions with sterile peptone water. To determine the growth of different isolates in the samples, aliquots of the dilutions were cultured on plates of de Man Rogosa and Sharpe (MRS) agar (Oxoid, Basingstoke, UK) for the growth of lactic acid bacteria, polymyxin pyruvate egg-yolk mannitol–bromothymol blue (PEMBA) agar (Oxoid) for the growth of *Bacillus* spp., MacConkey (MCK) agar (BioMèrieux, Marcy l’Etoile, France) for the growth of Gram-negative bacteria, Columbia nalidixic acid (CNA) agar (BioMèrieux) for the growth of staphylococci, streptococci, enterococci, corynebacterial, and related Gram-positive bacteria, and Sabouraud chloramphenicol dextrose (SDC) agar (BioMèrieux) for the growth of yeasts and molds. All of the plates were incubated at 37 °C for 48 h. The isolates obtained from the MRS plates were identified by means of 16S rDNA gene amplification and sequencing using a procedure that had been described previously [18]. All of the isolates identified as *L. salivarius* were genotyped by using a Random Amplification of Polymorphic DNA (RAPD) protocol [19]. In addition, the evolution of the pH and bacterial counts during cold storage for 42 days was assessed in four containers belonging to six different batches. 

### 2.3. Study Design and Participants

The study was conducted in an elderly nursing home located in Moralzarzal (Madrid, Spain). This establishment had 47 residents immediately before the pandemic, but the population was halved when it reached the location: 18 residents died in a period of a few weeks (4 at a hospital and 14 at the nursing home, including 10 with typical COVID-19-associated symptoms), but none of them were PCR tested at that time; therefore, none of them officially died because of this disease. When the 29 surviving residents were tested for SARS-CoV-2 by means of PCR, most of them were PCR-positive, and this was the case for most of the staff members as well. 

In this context, the trial was designed to include all of the residents as long as (a) informed consent was obtained from the participants or their legal representatives, (b) they were not fed by parenteral nutrition exclusively, and/or (c) they were not allergic to cow’s milk proteins (because the probiotic was delivered in a dairy food matrix). A total of 25 residents aged 74–98 met these criteria and started the trial. Starting at day 0, the residents consumed a fermented dairy product daily (125 g; ~9.3 log_10_ CFU of *L. salivarius* MP101 per product) for 4 months. Two samples (nasal wash and feces) were collected from each patient at recruitment (day 0) and at the end of the study (day 120). The nasal wash was obtained using a standardized protocol [20]. Aliquots of the samples were stored at −80 °C until the immunological analyses were performed. 

Some demographic and health-related data (gender, age) were recorded at recruitment, while other health-related data (SARS-CoV-2 status, body mass index (BMI), type of diet, concomitant diseases, and medication) were recorded both at recruitment and at the end of the study (Table 1; Supplementary Table S1). The staff of the nursing home recorded compliance with intake of the study product, and the minimum compliance rate (% of days ingesting the study product) was set at 86%. This study was conducted according to the guidelines determined in the Declaration of Helsinki and was approved by the Ethics Committee of the Hospital Clínico San Carlos (Madrid, Spain) (protocol: CEIC 20/263-E_COVID; date of approval: 01/04/2020, act 4.1/20). The trial was registered in the ClinicalTrials.gov database (identifier: NCT04922918).

### 2.4. Measurement of Functional, Cognitive and Nutritional Status of the Participants

The functional, cognitive, and nutritional status of the participants were assessed at recruitment and at the end of the study. The Barthel index (BI) was used to measure the functional status of ten basic activities of daily living (feeding, bathing, dressing, grooming, defecation, urination, toilet use, transfer, mobility, and stairs). The total score ranges from 0 (total dependent living) to 100 (total independent living) [21]. Cognitive, affective, and mood status was assessed using the Global Deterioration Scale and the Functional Assessment Staging (GDS/FAST) procedure, which includes seven stages, ranging from normal aging (stage 1) to severe dementia (stage 7) [22]. Finally, nutritional status was evaluated using the Mini Nutritional Assessment (MNA) score [23], which ranges from 0 to 30 points (≥24: no nutritional problems; 17–23.5: vulnerable to malnutrition; <17: malnourished). 

### 2.5. Immunoprofiling of the Nasal and Fecal Samples

Nasal samples (1 mL) were centrifuged, and the supernatants were used for the immunological assays. Fecal samples were prepared as described previously [24]. The concentrations of a wide array of inflammation-related immune factors (APRIL/TNFSF13, BAFF/TNFSF13B, Chitinase 3-like 1, IFNα, IFNβ, IFNγ, IL2, IL8, IL10, IL11, IL12p40, IL12p70, IL19, IL20, IL22, IL26, IL27p28, IL28/IFNλ2, IL29/IFNλ1, IL32, IL34, IL35, LIGHT/TNFSF14, MMP-1, MMP-2, MMP-3, osteocalcin, osteopontin, pentraxin 3, TSLP, TWEAK/TNFSF12, gp130/sIL-6Rb, sCD30/TNFRSF8, sCD163, sIL-6Ra, sTNF-R1, and sTNF-R2) were determined using the Bio-Plex Pro Human Inflammation Assay kit (Bio-Rad) in the Bio-Plex 200 instrument (Bio-Rad, Hercules, CA, USA). Every assay was run in duplicate, and standard curves were performed for each analyte.

### 2.6. Statistical Analysis

Data distribution was evaluated using Shapiro–Wilk normality tests. Data with normal distribution were expressed as the mean and a 95 confidence interval (95% CI), and data with non-normal distribution were expressed as the median and interquartile range (IQR). Microbiological data recorded as CFU per container were log_10_ transformed before analyses. Kruskal–Wallis tests were used to determine if there were differences in the bacterial concentration and pH values among the batches at each sampling time. Friedman’s non-parametric repeated measures comparisons followed by post hoc Nemenyi tests were applied to evaluate differences in these two parameters when different sampling times were compared. In the case of the parameters measured before and after the trial, differences in the normally distributed data were assessed using paired t-tests, and differences in non-normally distributed data were assessed using Wilcoxon signed-rank tests (library {PairedData version 1.1.1}), respectively. The significance level for the tests was declared at *p* < 0.05. Statistical analyses were performed with R-project software, version 4.0.3 (R-project, http://www.r-project.org (accessed on 29 April 2021)). 

## 3. Results

### 3.1. Concentration, Purity and Stability of L. salivarius MP101 in the Study Product

**Figure 1:**
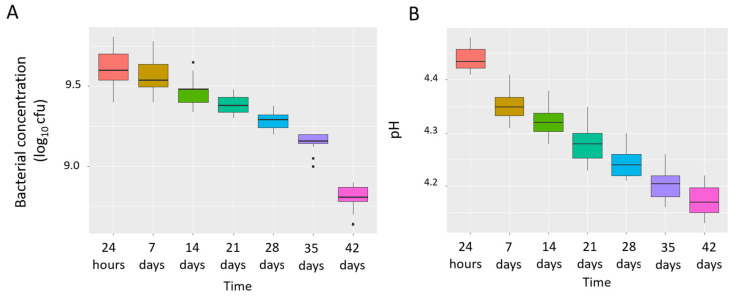
Box plots showing MRS bacterial concentration (log_10_ CFU/container) (**A**) and pH (**B**) values of the dairy products over time. The boxes represent the values of the interquartile ranges, with the median represented as a line. Outliers are represented as dots.

The bacterial concentration (CFU) per container (125 g) and the pH of the dairy products were measured at 24 h and at 7, 14, 21, 28, 35 and 42 days after the reception of each batch. Microbial growth was only observed on the MRS plates, and no growth was detected on the remaining media (PEMBA, MCK, CNA, SDC) used to control the purity of the developed product. Median (IQR) values of the MRS counts decreased progressively from 9.60 [9.54–9.70] log_10_ CFU/container (125 g) at 24 h to 8.81 [8.78–8.87] log_10_ CFU/container at day 42 (Figure 1A). The median (IQR) of the pH value after manufacture (24 h) was 4.43 [4.42–4.46] and dropped to 4.17 [4.17–4.20] after 42 days (Figure 1B). Both the MRS bacterial counts and the pH values of the probiotic product decreased slowly throughout the cold storage period (Figure 1), and the differences were only statistically significant when the values obtained at 24 h were compared to those observed after 28, 35, and 42 days (*p* < 0.05 for MRS bacterial counts and pH values, respectively; Friedman’s non-parametric repeated measures comparisons followed by post hoc Nemenyi tests). The MRS counts were above 9 log_10_ CFU/container (concentration considered as the minimum threshold dose for the trial) until week 5. Consequently, each batch was used for a maximum of 5 weeks after production. 

No differences in either the bacterial counts or in the pH were observed between the batches within each sampling time (*p* > 0.175 and *p* > 0.353, respectively; Kruskal–Wallis tests), indicating the reproducibility of the manufacturing process.

Only one colony morphology (compatible with that of *L. salivarius* MP101) was observed on the MRS plates. The identification of the isolates revealed that all of them belonged to the species *L. salivarius* (>99% identity in the sequence). RAPD genotyping of the *L. salivarius* isolates revealed that all of them shared the same RAPD profile as the inoculated strain (*L. salivarius* MP101).

### 3.2. Evolution of the COVID-19, Functional, Cognitive and Nutritional Status of the Participants

A total of 22 out of the 25 recruited participants finished the trial (Table 1). One participant died during the first weeks of the trial, but she already was in a terminal stage when the trial started (98 years of age; BI: 0; GDS/FAST: 7; MNA: 12.5; skin cancer, COVID-19-positive), while the other two participants were moved from the nursing home to a relative’s house. 

All of the recruited elderly patients suffered from several, often severe, diseases and were polymedicated (Supplementary Table S1). In addition, a high percentage of the participants (81%) were SARS-CoV-2-positive (mild or asymptomatic disease) at the beginning of the trial; however, all of them were negative at day 120. Interestingly, none of them became infected or re-infected with SARS-CoV-2 throughout the trial. All of the participants received the *Pfizer*-BioNTech COVID-19 vaccine on 19th January 2021 (first dose) and 9th February 2021 (second dose). 

In relation to the functional, cognitive, and nutritional status of the participants, there was no change in the GDS/FAST scores of each patient before and after the trial (Table 2). In contrast, the mean or median values for the BI index and the MNA score improved significantly from day 0 (36 and 20.70, respectively) to day 120 (42 and 22.63, respectively) (Table 2).

### 3.3. Evolution of the Immunological Parameters in the Nasal Samples

In relation to the nasal immunological profiles, the frequency of detection was high (>75% of the samples at both sampling times) for the following immune factors: BAFF/TNFSF13B (which was the only one detected in 100% of the nasal samples), APRIL/TNFSF13, chitinase 3-like 1, IL8, IL32, osteopontin, pentraxin 3, TWEAK/TNFSF12, and gp130/sIL-6Rb (Table 3). In contrast, IFNγ, IL2, IL10, IL20, IL27p28, IL28A/IFNλ2, and IL29A/IFNλ1 were not detected in any of the samples. There were statistically significant differences in the prevalence of several immune factors when samples from day 0 and day 120 were compared. The prevalence decreased over time for IL11, IL12 p70, LIGHT/TNFSF14, MMP-1, TSLP, sTNF-R1, and sTNF-R2, while they increased in the case of pentraxin 3 (*p* ≤ 0.03; Fisher exact tests) (Table 3). 

BAFF/TNFSF13B, APRIL/TNFSF13, and chitinase 3-like 1 were the immune compounds present in the highest concentrations (μg/L range) in comparison with the rest of the detected factors (ng/L range) (Table 3). The concentrations for BAFF/TNFSF13B, APRIL/TNFSF13, 1, IL8, IL32, osteopontin, and sTNF-R1 were lower at the end of the trial (day 120) compared to the starting time (day 0), and the opposite was observed for chitinase 3-like 1, pentraxin 3, IL19, and IL35 (*p* ≤ 0.04; paired t-tests or Wilcoxon signed-rank tests) (Table 3). 

### 3.4. Evolution of the Immunological Parameters in the Fecal Samples

In relation to the fecal immunological profiles, the detection frequency was high (>75% of the samples at both sampling times) in a narrower spectrum of immune factors: BAFF/TNFSF13B (the only one detected in 100% of the fecal samples), chitinase 3-like 1, IL32, osteopontin, and gp130/sIL-6Rb (Table 4). Only IL2, IL10, and IL29A/IFNλ1 were not detected in any of the fecal samples. The prevalence decreased from day 0 and day 120 for sTNF-R1 and sTNF-R2, while it increased for IFNα2 (*p* ≤ 0.048; Fisher exact tests) (Table 4). 

Similar to the nasal samples, the immunological compounds BAFF/TNFSF13B, APRIL/TNFSF13, and chitinase 3-like 1 had the highest concentrations (μg/L range) in the fecal samples (Table 4). The concentrations of BAFF/TNFSF13B, APRIL/TNFSF13, chitinase 3-like 1, IL32, IL34, gp130/sIL-6Rb, sTNF-R1, and sTNF-R2 decreased from day 0 to day 120 time, while those of pentraxin 3, MMP2, IL19, IL35, and sCD163 increased (*p* ≤ 0.040; paired t-tests or Wilcoxon signed-rank tests) (Table 4).

## 4. Discussion

Although COVID-19 is a viral infection, emerging evidence indicates that the composition of the respiratory and gastrointestinal microbiotas may be associated with a higher or lower severity of the disease through their immunomodulation and barrier functions [25,26]. As a consequence, the use of probiotics enhancing such functions has been postulated in the context of the current pandemic [10]. So far, two studies have found a positive effect of a probiotic product that shortens the duration of diarrheal episodes and decreases the risk of respiratory failure [14] or the risk of death [15]. In this study, a *L. salivarius* strain specifically selected for the prevention of a viral respiratory infection (RSV-associated bronchiolitis) was tested for the potential functional, nutritional, and immunological benefits that might it provide to a highly vulnerable elderly population living in a nursing home with a high rate of SARS-CoV-2 positive individuals. 

In order to minimize the management efforts in the nursing home, the strain was administered as a dairy product, which involved a previously mentioned development step. Microbiological analyses of six batches showed that the fermented product only contained *L. salivarius* MP101 and contained the appropriate amount of viable probiotic cells considered valid for the assay (≥9 log_10_ CFU/container) for five weeks, provided that the probiotic dairy product was stored at refrigeration temperatures. *L. salivarius* is a species that contains strains with a high probiotic potential, as assessed by in vitro and in vivo assays and clinical trials [27,28,29,30]. However, very few strains are commercially available because of their short stability at room temperature. Since the shelf life of fermented milks is usually 4 weeks under refrigeration conditions, the development of a *L. salivarius*-containing fermented milk may provide a suitable format to provide these benefits to consumers, especially for collectivities in which physical or mental disabilities are widespread, such as in nursing homes for the elderly.

In this study, probiotic intake led to an improvement of the BI and the MNA score. This is also a relevant finding since studies that have been performed so far have revealed that there is a significant worsening of functional ability in daily living activities in COVID-19 patients after infection, regardless of the scale applied, and that older patients are associated with the worse results [31]. Some of these studies were performed using the same tool (BI) employed in our work [32,33,34]. Similarly, previous studies have shown that there is a high nutritional risk among COVID-19 patients and that this risk is higher among the elderly [35]. The strengthening of nutritional support during and after COVID-19 seems essential, especially for older adults with diabetes mellitus [36]. Interestingly, the nutritional status (as assessed using the BI) of all the diabetic elderly that participated in our trial improved after probiotic consumption. 

In relation to the immunological profiles, the concentrations of a few immune factors changed significantly after the trial. Among them, the decrease in the concentrations of BAFF/TNFSF13B, APRIL/TNFSF13, IL-8, IL31, osteopontin, sTNF-R1, and sTNF-R2 and the increase in those of chitinase 3-like 1, IL19, IL35, and pentraxin 3 seemed particularly relevant since most of them were present in a (relatively) high percentage in both types of samples. The concentration of BAFF/TNFSF13B was significantly higher in both the nasal and fecal samples before the probiotic intervention. Most participants were SARS-CoV-2-positive when recruited and, in addition, some of them suffered from chronic respiratory diseases. BAFF is highly expressed in the airways during infectious and inflammatory processes, including in cystic fibrosis and RSV infections, and as a result, its concentration increases in samples of bronchoalveolar and nasal lavage fluid obtained from these patients [17,37,38,39]. Increased BAFF levels in fecal samples seems to be a feature of gut inflammation and, in fact, this immune factor may be used as a biomarker for inflammatory bowel diseases [40]. RSV-associated bronchiolitis has also been linked to increased IL8 and sTNF.R1 levels in nasal samples [17,41,42].

The results presented in this work are focused on the nasal and fecal samples from an elderly population living in a nursing home. This is a novelty in the field, but at the same time, this also limits comparison with results obtained by other authors. The nasal environment plays a central role in the acquisition and modulation of SARS-CoV-2 infection, but information on the immune responses developed in the upper respiratory tract against this virus is very scarce [43]. It must be taken into account that COVID-19-related immunoprofiling has mostly been limited to the blood samples of hospitalized patients during acute phase infections and that the blood and mucosal compartments may behave differently. As an example, high blood levels of pentraxin 3, IL19, and chitinase 3-like 1 have been associated with poor outcome in SARS-CoV-2 patients [44,45,46]. In contrast, the results of this study showed that the nasal and fecal levels of these immune compounds increased after the probiotic trial although the final values were much lower than those related to poor outcomes in blood samples. In the mucosal compartments, their increases may contribute to the clearance of or resistance to pathogens, to tissue repair, and to a decrease of the inflammatory state that usually characterizes the respiratory and gastrointestinal tracts in the elderly [47]. Such health-promoting activities in the respiratory and gut environments may explain the improvements in the functional and nutritional scores observed after the probiotic intervention.

This study faces some limitations, including the low number of participants and, due to ethical reasons, the absence of a placebo group. Our results suggest that some immune factors could be used as nasal or fecal biomarkers of probiotic efficacy in SARS-CoV-2-affected elderly, but this has to be confirmed in future studies. However, despite the heterogeneity of the recruited participants, a general improvement in the functional, nutritional, and immunological status was observed after the trial, which is uncommon in elderly nursing homes that have been highly affected by the COVID-19 pandemic. In conclusion, *L. salivarius* MP101 may be a promising strain for use as an aid for improving or maintaining health in this highly vulnerable population. 

## Figures and Tables

**Table 1:** Main characteristics of the elderly population that completed the trial (*n* = 22).

Participant Characteristic	Mean (95% CI) or *n* (%)
**Age (years)**	84.95 (81.41–88.49)
**Gender**	data
Male	11 (50%)
Female	11 (50%)
**Type of diet**	
Normal	10 (45%)
Normal/Diabetes	7 (32%)
Pureed (turmix) foods	5 (23%)
**BMI (kg/m^2^)**	
Day 0	24.82 (22.88–26.751)
Day 120	24.71 (23.27–26.16)
**SARS-CoV-2 (positive PCR/total)**	
Day 0	18/22 (81%)
Day 120	0/22 (0%)

BMI, body mass index.

**Table 2:** Assessment of the functional, cognitive, and nutritional status of the participants at the start and at the end of the trial. Values are expressed as mean (95% CI) or median (IQR).

Index or Score ^1^	Day 0	Day 120	*p*-Value
BI	36.00 [22.75–80.50]	42.00 [25.25–84.25]	0.022
GDS/FAST	4.00 [3.25–6.00]	4.00 [3.25–6.00]	-
MNA	20.70 (18.80–22.60)	22.63 (21.20–24.06)	0.001

^1^ BI, Barthel index; GDS/FAST, Global Deterioration Scale and Functional Assessment Staging; MNA, Mini Nutritional Assessment.

**Table 3:** Frequency of detection (%) and concentration of immune factors in nasal samples from the recruited participants before (day 0) and at the end (day 120) of the trial.

	Day 0		Day 120			
Immune Factor	Frequency of Detection	Median (IQR) or Mean (95%CI)	Frequency of Detection	Median (IQR) or Mean (95%CI)	*p*-Value ^a^	*p*-Value
APRIL/TNFSF13, μg/L	22 (100%)	**7.35 [3.52–9.47]**	21 (96%)	**6.80 [3.00–8.35]**	>0.99	**<0.001 ^n^**
BAFF/TNFSF13B, μg/L	22 (100%)	**2.76 (2.35–3.16)**	22 (100%)	**0.89 (0.79–0.99)**	>0.99	**<0.001 ^s^**
Chitinase 3-like 1, μg/L	17 (77%)	**0.10 [0.68–1.31]**	17 (77%)	**0.95 [0.78–1.26]**	>0.99	**0.040 ^n^**
IFNα2, ng/L	2 (9%)	20.05	2 (9%)	20.40 [19.92–20.87]	>0.99	-
IFNβ, ng/L	4 (18%)	2.03 [1.90–2.28]	1 (5%)	1.47	0.4	-
IL8, ng/L	18 (82%)	**2.56 [2.42–3.30]**	17 (77%)	**0.49 [0.27–0.60]**	>0.99	**<0.001 ^n^**
IL11, ng/L	**7 (32%)**	0.66 [0.52–0.75]	**0 (0%)**	0	**0.008**	-
IL12p40, ng/L	4 (18%)	2.32 [2.14–2.65]	0 (0%)	0	0.1	-
IL12p70, ng/L	**6 (27%)**	0.13 [0.12–0.13]	**0 (0%)**	0	**0.02**	-
IL19, ng/L	8 (36%)	**1.55 [0.96–2.45]**	15 (68%)	**4.76 [3.85–9.42]**	0.068	**0.008 ^n^**
IL22, ng/L	2 (9%)	2.77	2 (9%)	2.76	>0.99	-
IL26, ng/L	0 (0%)	0	1 (5%)	0.46	>0.99	-
IL32, ng/L	18 (82%)	**6.37 (5.32–7.43)**	17 (77%)	**3.10 (2.29–3.91)**	>0.99	**<0.001 ^s^**
IL34, ng/L	6 (27%)	20.71 [18.72–22.88]	4 (18%)	12.5 [11.31–13.17]	0.72	-
IL35, ng/L	8 (36%)	**0.64 [0.52–1.09]**	8 (36%)	**1.14 [1.07–2.28]**	>0.99	**0.008 ^n^**
LIGHT/TNFSF14, ng/L	**13 (59%)**	1.19 (0.93–1.44)	**5 (23%)**	0.63 (0.00–1.35)	**0.03**	0.684 ^s^
MMP-1, ng/L	**6 (27%)**	17.60 [15.00–18.97]	**0 (0%)**	0	**0.02**	-
MMP-2, ng/L	6 (27%)	34.69 [24.06–50.05]	2 (9%)	14.54	0.24	-
MMP-3, ng/L	4 (18%)	31.65 [30.05–34.12]	0 (0%)	0	0.1	-
Osteocalcin, ng/L	3 (14%)	2.24 [1.52–2.25]	1 (5%)	2.97	0.61	-
Osteopontin, ng/L	22 (100%)	**81.22 (69.51–92.93)**	18 (82%)	**61.74 (49.02–74.47)**	0.1	**<0.001 ^s^**
Pentraxin 3, ng/L	**7 (77%)**	**0.71 [0.63–2.44]**	**22 (100%)**	**2.75 [2.28–4.22]**	**<0.001**	**0.016 ^n^**
TSLP, ng/L	**17 (77%)**	0.73 (0.50–0.96)	**4 (18%)**	0.42 (0.00–1.34)	**<0.001**	0.075 ^s^
TWEAK/TNFSF12, ng/L	17 (77%)	2.15 [0.72–3.41]	19 (86%)	2.28 [0.80–3.44]	0.69	0.579 ^n^
gp130/sIL-6Rb, ng/L	22 (100%)	155.88 (125.89–185.88)	20 (91%)	157.79 (123.62–191.96)	0.48	0.154 ^s^
sCD30/TNFRSF8, ng/L	4 (18%)	40.84 [14.45–65.42]	4 (18%)	44.02 [19.45–68.21]	>0.99	-
sCD163, ng/L	0 (0%)	0	4 (18%)	2.88 [2.22–3.69]	0.1	-
sIL-6Ra, ng/L	4 (18%)	10.75 (0–26.46)	8 (36%)	11.47 (1.17–21.76)	0.31	0.351 ^s^
sTNF-R1, ng/L	**21 (96%)**	**34.64 (22.76–46.51)**	**13 (59%)**	**13.79 (2.58–25.01)**	**0.009**	**<0.001 ^s^**
sTNF-R2, ng/L	**21 (96%)**	12.82 (9.43–16.21)	**6 (27%)**	4.50 (1.62–7.38)	**<0.001**	0.212 ^s^

^a^ Fisher exact tests were used to evaluate differences in the prevalence/detection rates of the analyzed parameters. ^n^ Wilcoxon signed-rank tests were used to evaluate differences in the concentration of the analyzed parameters with a non-normal distribution. ^s^ Paired t-tests were used to evaluate differences in the concentration of the analyzed parameters with a normal distribution. Bold font indicates values having statistically significant differences.

**Table 4:** Frequency of detection (%) and concentration of immune factors in fecal samples from the recruited participants before (day 0) and at the end (day 120) of the trial period.

	Day 0		Day 120			
Immune Factor	Frequency of Detection	Median (IQR) or Mean (95%CI)	Frequency of Detection	Median (IQR) or Mean (95%CI)	*p*-Value ^a^	*p*-Value
APRIL/TNFSF13, μg/L	12 (55%)	**1.72 (1.22–2.23)**	11 (50%)	**1.48 (0.99–1.97)**	>0.99	**0.0111 ^s^**
BAFF/TNFSF13B, μg/L	22 (100%)	**1.21 [0.96–1.51]**	22 (100%)	**0.49 [0.31–0.68]**	>0.99	**<0.001 ^n^**
Chitinase 3-like 1, μg/L	21 (95%)	0.35 [0.21–0.56]	22 (100%)	0.31 [0.20–0.44]	>0.99	0.0822 ^n^
IFNα2, ng/L	**0 (0%)**	0	**5 (23%)**	15.56 [12.12–17.87]	**0.048**	-
IFNβ, ng/L	6 (27%)	2.72 (1.52–3.92)	9 (41%)	2.82 (1.81–3.83)	0.52	0.407 ^s^
IFNγ, ng/L	4 (18%)	5.42 [4.09–5.81]	0 (0%)	0	0.100	-
IL8, ng/L	8 (36%)	3.79 [2.20–6.34]	7 (32%)	1.15 [0.51–2.27]	>0.99	-
IL11, ng/L	5 (23%)	0.36 (0.24–0.49)	5 (23%)	0.36 (0.23–0.49)	>0.99	0.825 ^s^
IL12p40, ng/L	8 (36%)	4.99 (1.89–8.09)	9 (41%)	4.29 (1.59–6.98)	>0.99	0.845 ^s^
IL12p70, ng/L	11 (50%)	0.21 (0.11–0.31)	10 (45%)	0.22 (0.09–0.35)	>0.99	0.823 ^s^
IL19, ng/L	7 (32%)	**1.60 [1.19–2.37]**	14 (64%)	**4.05 [3.54–4.88]**	0.21	**0.016 ^n^**
IL20, ng/L	1 (5%)	0.32	0 (0%)	0	>0.99	-
IL22, ng/L	8 (36%)	2.37 (1.21–3.54)	10 (45%)	1.39 (0.66–2.12)	0.75	0.079 ^s^
IL26, ng/L	11 (50%)	1.28 (0.41–2.14)	12 (55%)	0.91 (0.41–1.41)	>0.99	0.675 ^s^
IL27p28, ng/L	9 (41%)	0.55 [0.44–3.76]	8 (36%)	0.37 [0.21–0.87]	>0.99	0.813 ^n^
IL28A/IFNλ2, ng/L	3 (14%)	4.52 [4.225–4.78]	3 (14%)	0.73 [0.565–1.91]	>0.99	-
IL32, ng/L	21 (95%)	**6.50 [5.89–8.09]**	20 (91%)	**3.47 [2.67–5.10]**	>0.99	**<0.001 ^n^**
IL34, ng/L	16 (73%)	**40.57 (30.55–50.59)**	16 (73%)	**19.19 (11.03–27.35)**	>0.99	**0.002 ^s^**
IL35, ng/L	16 (73%)	**7.79 [4.23–10.23]**	16 (73%)	**10.33 [6.39–15.56]**	>0.99	**0.005 ^n^**
LIGHT/TNFSF14, ng/L	12 (55%)	1.39 (0.81–1.98)	14 (64%)	0.87 (0.43–1.31)	0.750	0.228 ^s^
MMP-1, ng/L	7 (32%)	26.85 [22.37–89.33]	3 (14%)	6.30 [3.96–20.34]	0.280	-
MMP-2, ng/L	10 (45%)	**50.10 (23.35–76.86)**	6 (27%)	**70.08 (44.45–95.72)**	0.340	**0.016 ^s^**
MMP-3, ng/L	4 (18%)	36.61 [29.21–59.21]	1 (5%)	23.39	0.340	-
Osteocalcin, ng/L	2 (9%)	3.27	4 (18.18%)	2.58 [1.99–3.71]	0.660	-
Osteopontin, ng/L	22 (100%)	88.61 [75.96–100.07]	21 (95%)	80.70 [74.87–94.65]	>0.99	0.128 ^n^
Pentraxin 3, ng/L	12 (55%)	**0.97 (0.39–1.55)**	22 (100%)	**2.84 (2.38–3.30)**	0.34	**<0.001 ^s^**
TSLP, ng/L	19 (86%)	0.94 [0.73- 1.94]	16 (73%)	0.86 [0.60–1.78]	0.450	0.551 ^n^
TWEAK/TNFSF12, ng/L	21 (95%)	3.19 [2.32–4.23]	21 (95%)	3.69 [2.62–5.01]	>0.99	0.063 ^n^
gp130/sIL-6Rb, ng/L	20 (91%)	**79.57 (67.48 91.65)**	20 (91%)	**71.80 (60.79–82.81)**	>0.99	**<0.001 ^s^**
sCD30/TNFRSF8, ng/L	11 (50%)	22.41 [13.51–40.15]	13 (59%)	14.87 [4.45–37.20]	0.76	0.831 ^n^
sCD163, ng/L	4 (18%)	**5.07(3.72–6.42)**	5 (23%)	**6.57 (5.72–7.42)**	>0.99	**0.037 ^s^**
sIL-6Ra, ng/L	1 (5%)	10.03	6 (27%)	2.69 [2.35–3.2625]	0.090	-
sTNF-R1, ng/L	**22 (100%)**	**29.62 [19.33–36.86]**	**16 (73%)**	**4.98 [1.13–9.14]**	**0.020**	**<0.001 ^n^**
sTNF-R2, ng/L	**22 (100%)**	**19.72 (15.22–24.23)**	**14 (64%)**	**3.25 (1.18–5.33)**	**0.003**	**<0.001 ^s^**

^a^ Fisher exact tests were used to evaluate differences in the prevalence/detection rates of the analyzed parameters. ^n^ Wilcoxon signed-rank tests were used to evaluate differences in the concentration of the analyzed parameters with a non-normal distribution. ^s^ Paired *t*-tests were used to evaluate differences in the concentration of the analyzed parameters with a normal distribution. Bold font indicates values having statistically significant differences.

## Data Availability

Data supporting the reported results can be found in Tables S1 and S2.

## Supplementary Materials

The following are available online at https://www.mdpi.com/article/10.3390/foods10092149/s1, Table S1: Demographic and health-related data of the participants that completed the trial. Table S2: Immunological data from the fecal and nasal samples obtained at days 0 and 120 from each patient that completed the trial.
